# Arterial dissections: Common features and new perspectives

**DOI:** 10.3389/fcvm.2022.1055862

**Published:** 2022-12-06

**Authors:** Monique Bax, Valentin Romanov, Keerat Junday, Eleni Giannoulatou, Boris Martinac, Jason C. Kovacic, Renjing Liu, Siiri E. Iismaa, Robert M. Graham

**Affiliations:** ^1^Victor Chang Cardiac Research Institute, Darlinghurst, NSW, Australia; ^2^UNSW Medicine and Health, UNSW Sydney, Kensington, NSW, Australia; ^3^St. Vincent’s Hospital, Darlinghurst, NSW, Australia; ^4^Icahn School of Medicine at Mount Sinai, Cardiovascular Research Institute, New York, NY, United States

**Keywords:** arterial dissection, aortic dissection, spontaneous coronary artery dissection (SCAD), cervical artery dissection (CeAD), TGF-β, extracellular matrix, vascular smooth muscle cells (VMSCs), endothelial cells (ECs)

## Abstract

Arterial dissections, which involve an abrupt tear in the wall of a major artery resulting in the intramural accumulation of blood, are a family of catastrophic disorders causing major, potentially fatal sequelae. Involving diverse vascular beds, including the aorta or coronary, cervical, pulmonary, and visceral arteries, each type of dissection is devastating in its own way. Traditionally they have been studied in isolation, rather than collectively, owing largely to the distinct clinical consequences of dissections in different anatomical locations – such as stroke, myocardial infarction, and renal failure. Here, we review the shared and unique features of these arteriopathies to provide a better understanding of this family of disorders. Arterial dissections occur commonly in the young to middle-aged, and often in conjunction with hypertension and/or migraine; the latter suggesting they are part of a generalized vasculopathy. Genetic studies as well as cellular and molecular investigations of arterial dissections reveal striking similarities between dissection types, particularly their pathophysiology, which includes the presence or absence of an intimal tear and vasa vasorum dysfunction as a cause of intramural hemorrhage. Pathway perturbations common to all types of dissections include disruption of TGF-β signaling, the extracellular matrix, the cytoskeleton or metabolism, as evidenced by the finding of mutations in critical genes regulating these processes, including *LRP1*, collagen genes, fibrillin and TGF-β receptors, or their coupled pathways. Perturbances in these connected signaling pathways contribute to phenotype switching in endothelial and vascular smooth muscle cells of the affected artery, in which their physiological quiescent state is lost and replaced by a proliferative activated phenotype. Of interest, dissections in various anatomical locations are associated with distinct sex and age predilections, suggesting involvement of gene and environment interactions in disease pathogenesis. Importantly, these cellular mechanisms are potentially therapeutically targetable. Consideration of arterial dissections as a collective pathology allows insight from the better characterized dissection types, such as that involving the thoracic aorta, to be leveraged to inform the less common forms of dissections, including the potential to apply known therapeutic interventions already clinically available for the former.

## Arterial features define vulnerability to dissections

**FIGURE 1:**
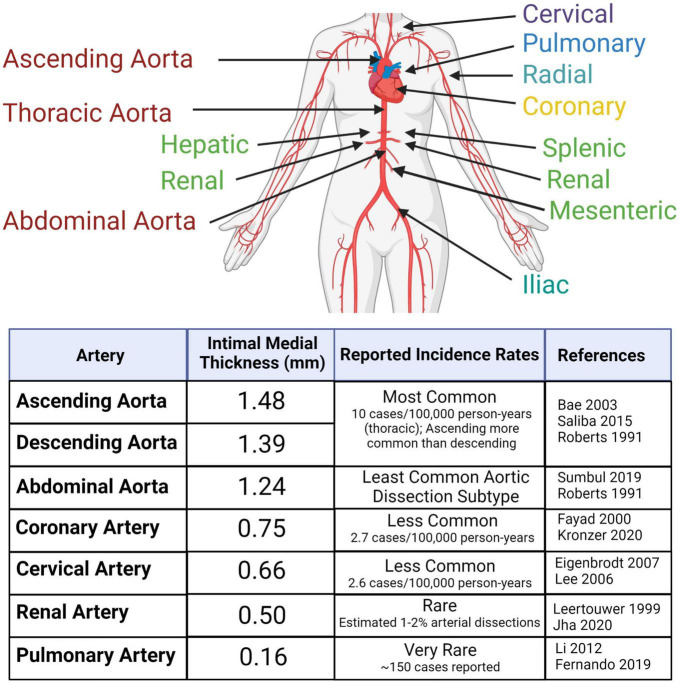
Arterial dissections are reported in large- and medium-sized arteries throughout the body at varying frequencies within the population. The risk of dissection varies with sex and age. Reported incidences correlate with average intimal medial thickness. Created with BioRender.com.

An arterial dissection is a structural failure of an arterial wall that results in an intramural bleed, which forms an intramural hematoma (IMH) that dissects the vessel wall. As the IMH expands it causes the ipsilateral vessel wall to bulge into the vessel lumen toward the contralateral wall, which in smaller diameter vessels leads to obstruction of blood flow. This obstruction prevents tissue perfusion causing ischemia and/or infarction. If the dissection is accompanied by an intimal tear, obstruction of the true lumen can be due to the IMH causing a thrombus that extends into and occludes the true lumen or can give rise to emboli that occlude distal arterial branches, resulting in micro-infarcts. The one exception to IMH-induced luminal occlusion is dissection of the aorta, which has a very large diameter, wherein the most concerning complication is not obstruction of blood flow or embolic events, but extension of the dissection into the pericardial space resulting in a hemopericardium that can causes pericardial tamponade, or extension of the dissection into a smaller diameter branch, such as a renal artery, resulting in renal ischemia or infarction. Dissections occur in medium and large arteries; with occurrences decreasing with diminishing arterial size (Figure 1). Clinically, dissections are often categorized by anatomical location. This localization predicates the medical specialty best-relating to the dissection – aortic and coronary artery dissections, which cause heart failure, are the focus of cardiologists; cervical dissections, which lead to migraines, or in severe cases, stroke, are the focus of neurologists. Despite the growth of vascular medicine as a specialty that bridges these anatomic regions, few reviews to date have considered these conditions collectively as a spectrum of diseases. Undeniable similarities between arterial dissections implicate common disease mechanisms. Aortic dissection, one of the best studied arterial dissection types, is associated with dysfunction of interlinked TGF-β signaling pathways, and disruption of extracellular matrix (ECM) structure, cytoskeletal function and vascular smooth muscle cell metabolism (1). Combining this research with increasing knowledge from other types of dissections and with heritable diseases commonly associated with arterial dissections will provide insight for better therapeutic intervention and prevention, which are severely lacking in many of these lesser understood dissection disorders.

**FIGURE 2:**
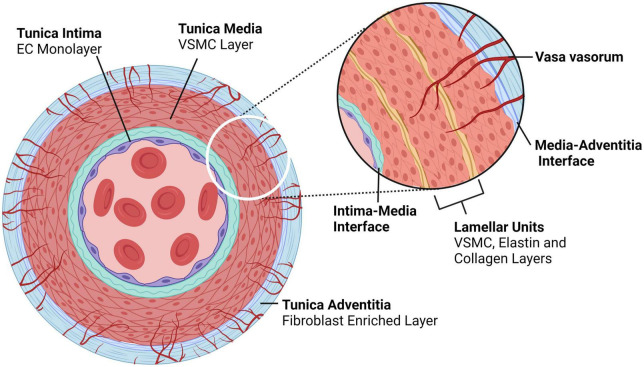
Typical architecture of medium to large, elastic and muscular arteries. Arteries comprise of three layers – the tunica intima is the innermost layer consisting of an endothelial monolayer; abluminal this monolayer is an ECM membrane located between the intima and the media, the latter being the second layer of the artery. The tunica media is concentrically layered with lamellar units of VSMCs bounded by elastin- and collagen-rich fibers. In larger arteries, the media is perfused by capillaries known as vasa vasorum, which enter the media through the outermost layer, the tunica adventitia; an ECM and fibroblast-rich layer. Created with BioRender.com.

Dissections are thought to originate in two of the three arterial layers – in the innermost layer, composed of an endothelial cell (EC) monolayer (*tunica intima;* intima), and in the thick muscular middle layer (*tunica media;* media) composed of concentric layers of vascular smooth muscle cells (VSMCs) and ECM that together form the lamellar unit (2). The outermost layer, an ECM coating (*tunica adventitia;* adventitia) is not a site of dissection-initiation (Figure 2).

These arterial layers vary anatomically. As muscular arteries branch further away from the heart their thickness decreases because of a decreasing number of lamellar units within the *tunica media.* This muscular layer provides the mechanical qualities – distensibility and elasticity – of a vessel. Without these properties, the vessel would lack compliance, resulting in excessive pressure within the vessel that would impair the ejection of blood from the heart. These large conduit arteries are also referred to as elastic arteries, owing to their high levels of elasticity facilitated by an abundance of elastin-rich ECM. As vessel size decreases, the amount of ECM, relative to VSMCs, is reduced. Medium-sized vessels are thus referred to as muscular arteries (or distributing arteries). As arteries branch further into resistance vessels, the media is further reduced (3). Dissections have not been reported in resistance vessels. Branching of arteries adds complexity in vascular structure, contributing to arterial microenvironments. For example, non-linear vessel morphology, including divergent junctions or bifurcations, exposes differing arterial regions to varying blood flow forces (shear stress).

### Elastic and muscular arteries require vasa vasorum

Larger arteries that exceed 29 lamellar units or are >5 mm in thickness (with the exception of the coronary arteries) have vasa vasorum, which are required for perfusion and oxygenation of the arterial wall (4). Functionally, vasa vasorum are end arteries, terminating close to the adventitial-medial layer and are drained by postcapillary venules (5, 6). The vasa vasorum capillary bed is highly irregular, forming kinks, twists, and outpouchings (7, 8). Typically, vasa vasorum perfuse only the outer two-thirds of the medial layer, the inner third being oxygenated by diffusion from the lumen. However, under pathological conditions, such as atherosclerotic plaque-formation where there is a barrier to oxygenation *via* luminal diffusion, vasa vasorum can extend inward toward the lumen to also perfuse the subendothelial *tunica media* (9, 10). While medial vasa vasorum are required for perfusion of the *media* in muscular arteries, such as the thoracic aorta, muscular veins and the pulmonary artery, thinner-walled vessels, such as the abdominal aorta, only have adventitial vasa vasorum, which are not essential for perfusion and integrity of the medial layer (11).

Flow in medial vasa vasorum has been largely understudied except in the aorta, so the physiology of vasa vasorum flow remains unclear. It is unknown, for example, if and to what extent flow in medial vasa vasorum is supported by their own VSMCs and/or pericytes, and whether or not flow in the vasa vasorum is dependent on compressive forces, such as peristaltic pressure of the media resulting from VSMC contraction (11, 12). The scale and structure of the vasa vasorum confer several interesting microfluidic properties: (1) Flow of blood through these vessels will mostly be laminar, as viscous forces dominate over inertial forces at these scales, as evident from **Reynolds Number** (Re, below), however, both eddies and to some extent turbulence can be induced following intimal failure, leading to flow disturbances;


$$R\,e=\frac{\rho \,v\,L}{\mu }$$

where ρ is fluid density, *v* is fluid velocity and *L* is vessel length, and μ is fluid viscosity.

(2) The **circumferential stress** (σ, below), derived from Laplace’s Equation, acts perpendicular to the radius of the lumen. For small vessels circumferential stress is relatively low, as it scales directly with internal pressure. This acts to dilate the vessel, the radius of the lumen and the thickness of the vessel wall (13, 14). Vasa vasorum lumens range from ∼2 μm to 329 μm, with an average luminal size of ∼40 μm (15). A vessel of this size should accommodate the pressure moving through an artery, at diastole, without failing. As such, failure of the vasa vasorum by pressure overload is unlikely, unless the surrounding environment no longer provides the required compressive forces;


$$\sigma =\frac{P\;\,r}{w}$$

where *P* is internal pressure, *r* is lumen radius and *w* is wall thickness.

Placing the above relationship into context, arteries on average, have an inner lumen that is 25 times larger than that of the vasa vasorum. To reduce stress within the arterial wall, the wall thickness must increase. The average arterial wall can be 150 times thicker than the average wall thickness of the vasa vasorum (assuming wall thickness of an average arteriole) (16).

(3) To a certain extent, the vasa vasorum can be expected to exhibit viscoelasticity to accommodate the sudden rise in pressure, which results in rapid, non-linear dilation of the lumen that tapers off if the pressure is maintained. The extent to which the vasa vasorum is compressed or dilated depends on the surrounding tissue, including the ECM and its constituents exhibit both viscoelastic and strain-stiffening behavior (17);

(4) The flow of blood through the vasa vasorum, from the adventitia to the media, scales directly with pressure, as given by the **Hagen–Poiseuille Equation** (below) and inversely with length of the vessel, i.e., the longer the vessel, the lower the flow rate (Q). The high degree of tortuosity of the vasa vasorum has several effects: while the vessels are better protected from longitudinal strains, the increase in vessel length increases the pressure required to drive blood from their entry point in the adventitia to their termination in the media and on to the postcapillary venules;


$$Q=\frac{\pi \,r^{4}\,△\,P}{8\,\mu \,L}$$

where, *r* is vessel radius, Δ*P* is pressure difference, μ is viscosity and *L* is vessel length.

(5) The localization of vasa vasorum in the outermost section of the media is proposed to be a function of the compressive pressure exerted by the surrounding matrix and the underlaying luminal pressure (18). The extent to which the vasa vasorum propagate axially through the media depends on the pressure drop across the vessel (△*P* = pressure at inlet – pressure at outlet) and the point at which luminal pressure overcomes the necessary pressure to drive blood throughout the length of the vessel (pressure at lumen > pressure at outlet).

Anatomical differences in vasa vasorum are evident. The coronary arteries, while relatively thin walled, are exposed to large cyclical deformations, owing to the movement of the heart. Cyclical compression of vasa vasorum significantly reduces flow rate within the vessels, reducing nutrient exchange and oxygen delivery. As such, it is unsurprising that the density of vasa vasorum in coronary arteries is higher than that of carotid or renal arteries and is almost 10 times greater than that of the femoral arteries. In the case of the femoral artery, a high density of vasa vasorum is not required as its vasa vasorum do not undergo cyclical compression and, as such, are able to maintain relatively constant blood flow with fewer branches and with slightly larger luminal diameters (∼22%) (19).

There are also structural and site-specific differences between the vasa vasorum of different arteries. The endothelial-surface-fraction (endothelial surface area/vessel wall volume) of the coronary artery vasa vasorum exceeds that of renal or femoral arteries by threefold, while the vascular-area-fraction (vasa vasorum area/vessel wall area) of the coronary artery vasa vasorum is roughly twice as high (20). Accordingly, the vasa vasorum within the coronary arteries are designed to withstand higher pressures, permitting greater perfusion than the vasa vasorum of other arteries. Disruption of blood flow through the vasa vasorum has been shown to increase tissue stiffness leading to detachment of the layers near the adventitial-medial border. This suggests that the vasa vasorum play a vital role in aortic wall integrity (15). As tissue stiffens, the artery is less efficient at dampening oscillations and pulsatility arising from systolic and diastolic phases of the heart, resulting in greater flow instability. This instability can be characterized by the **Womersley number** (α, below) representing the ratio of unsteady forces to viscous forces (21). The Womersley number is very small in the vasa vasorum (0.1), indicating that viscous forces dominate within these vessels and are well dampened from pulsatility. In contrast, main arteries are exposed to pulsatility that is approximately 100 times larger than for vasa vasorum (22). For example, the Womersley number for the ascending aorta, thoracic aorta, abdominal aorta and the femoral artery are 17.8, 12.1, 7.38, and 3.14, respectively (23).


α=Dm2⁢ρ⁢ 2⁢π⁢fμ

where *D*_m_ is the diameter of the vessel, *p* is fluid density, *f* is heart rate and μ is blood viscosity.

Theoretically, to allow vasa vasorum blood flow in the absence of a supporting muscular layer requires that the distending forces or hydrostatic pressure within the vasa vasorum exceed the compressive forces in the media (12, 24). The efficacy with which arteries maintain the appropriate level of distensibility and elasticity to allow appropriate flow – both at the tissue and cellular level – is subject to the appropriate ECM composition and, thus, physical properties, as well as structural, and metabolic proteins. Expression of these proteins is influenced by environmental factors, such as sex, aging, and lifestyle.

### Endothelial cells are the first layer of defense in arteries

The EC monolayer forming the intima is the first line of protection against infection and injury, acting as a selectively permeable barrier to prevent toxin- and pathogen-entry into tissues, and is an important interface between the immune system and its cognate perfused tissues. Critical to vascular function, ECs are one of the main factors governing vascular tone (25). EC communication with the underlying VSMCs modulates vasodilation *via* the gaseous messenger nitric oxide (NO) secreted following VSMC contraction. NO signaling by ECs has been shown to regulate not only VMSC contractility, but also ECM composition (26), as well as inhibiting platelet aggregation (27) and regulating VSMC metabolism and proliferation (28, 29).

Of particular relevance to arterial dissections, ECs also play a key role in mediating the response to injury. ECs and megakaryocytes are the only cells that produce the glycoprotein, von Willebrand factor (vWF), a carrier protein for factor VIII required for blood coagulation (30). Following damage to the intima, vWF binds to platelet membrane glycoproteins and exposes ECM to mediate platelet adhesion leading to clot formation. The majority (95%) of vWF is secreted constitutively by ECs, however, 5% is retained by ECs and stored in cellular vesicles known as Weibel–Palade bodies, ready for localized release if required (31). EC-derived extracellular vesicles are important in mediating VSMC activation. Extracellular vesicles derived from rat ECs that were subjected to serum depletion were found to alter the VSMC proteome by upregulating VSMC stress responses as well as cellular metabolism (32).

#### Endothelial cell identity is fluid

**FIGURE 3:**
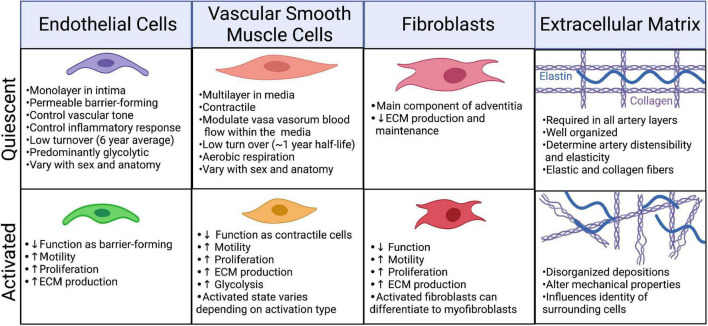
Phenotypic changes in the cellular and extracellular components of the artery in response to injury/infection. Created with BioRender.com.

The body is estimated to contain 2.54 ± 1.05 × 10^12^ ECs (33). Under physiological conditions quiescent ECs have an average lifespan of approximately 6 years and account for only ∼0.1% of the daily turnover of all cells (34, 35). Under conditions of growth or injury, ECs are activated, becoming “synthetic” or “secretory,” through upregulation of proliferative and secretory pathways [(36); Figure 3]. There are many subtypes of activated ECs, perhaps best defined by the inducing stimulus. Activation can be induced by blood vessel growth (angiogenesis) through growth factor signaling, such as by fibroblast growth factors (FGF) and vascular endothelial growth factor (VEGF), which upregulate proliferative, migratory, and invasive processes. Disturbances in blood flow force (shear stress), and inflammation (acute and chronic) can activate ECs. These activated cells upregulate inflammatory processes, including expression of inflammatory proteins such as VCAM1 and ICAM1, which promote immune cell infiltration (36, 37). Recent hypotheses propose that this inflammatory type of EC dysfunction occurs in response to SARS-CoV-2 infection (38).

#### Anatomy and sex influence endothelial cell identity

Quiescent ECs vary anatomically. A recent single cell sequencing analysis, which allowed the development of the first murine endothelial atlas, studied over 32,000 cells from 11 anatomical regions and identified 78 subclasses of ECs based on their transcriptome (39). This work confirmed earlier findings from DNA microarray analysis of ECs originating from different tissues and vessel types (40). These studies found that ECs group by vessel type, such as artery and vein, and also showed heterogeneity amongst cell types. Interestingly, ECs were found to have upregulation of pathways that were relevant to their anatomical origin – for example, ECs from the blood-brain barrier were upregulated for solute transport processes, while ECs derived from the liver and spleen, organs that filter and protect from pathogens, were upregulated for scavenging and immuno-regulating processes (40). Anatomical region and vascular type variations in ECs are not surprising given differences in the stimuli to which these cells are exposed. A study of human umbilical vein ECs from the same donor displayed variation in both their proteomes and lipidomes following exposure to differing shear stress (5 vs. 15 dynes/cm^2^) (41). These differences may represent potential anatomical vulnerabilities, rendering ECs from certain arteries more susceptible to particular stressors than others.

Endothelial cell populations also differ on the basis of sex. A comparison of umbilical vein EC transcriptomes between boy-girl twin pairs at birth indicated sex-specific differences in a number of pathways, such as enrichment in females for epithelial to mesenchymal transition and hypoxia, and in males the unfolded protein response and protein secretion (42). The same comparison was made in human adults (from the general population) and sex differences were again found, though some pathways were different, suggesting a sex hormone (rather than sex chromosome) effect whereby females had an enrichment for estrogen response pathways (early and late), while males had an increase in TGF-β pathway transcripts (42). These variables could contribute to risk of vascular diseases – including dissections – positively or negatively. Defining what is a healthy baseline EC expression profile will likely provide important insight into vascular diseases.

### Vascular smooth muscle cells are essential for structure in medium to large arteries

Under normal physiological conditions, VSMCs are contractile cells with a spindle-shaped morphology, reinforced by a well-ordered array of cytoskeletal proteins, including myosin heavy chain 11 (MYH11), α-smooth muscle actin (ACTA2), calponin (CNN1), and transgelin (TAGLN). Cellular contractility places a large metabolic demand on VSMCs. Yet, despite the advantage of oxidative phosphorylation in terms of energy economy, VSMCs use a combination of both oxidative phosphorylation and glycolytic metabolism to an almost equal extent when measured at rest (43). It is hypothesized that this acts to buffer energy depletion and prevent an ATP crisis (44), but persistent use of aerobic glycolysis (i.e., the Warburg effect) is also consistent with continued proliferation of VSMCs, even in adulthood. Despite these high energy demands, VSMCs are relatively long lived with a half-life of 270 – 400 days (45).

#### Vascular smooth muscle cell identity is also fluid

Like ECs, VSMCs can also undergo phenotypic switching from the physiological contractile phenotype to a highly proliferative synthetic cellular state in response to certain cues, such as injury (Figure 3). Under stress conditions, expression of cytoskeletal proteins, including MYH11, ACTA2, CNN1, and TAGLN, is reduced as cells switch from the contractile to a synthetic state. Earlier research defined synthetic VSMCs as cells that assume a more rounded morphology and have increased proliferative, secretory and migratory properties (46). Phenotype switching to the synthetic state coincides with a metabolic shift to increased reliance on glycolysis (47). Molecular triggers such as PDGF-BB, TGF-β, actin A, retinoids, angiotensin II, TNF-α, IGF-I, -II, endothelin-1, and NO as well as reactive oxygen species and a reduced glutathione (GSH) redox status, can all modulate the activation state of VSMCs (46, 48, 49). Activation can also be mediated by shear stress – synthetic VSMCs supplemented with media from ECs, even under low physiological stress (12 dynes/cm^2^), will upregulate contractile markers (50). VSMC phenotype has also been shown to be regulated epigenetically and the capacity for phenotypic modulation appears to be conserved across species, having been demonstrated in rats, pigs, cows, and humans (46, 51, 52).

Increased understanding of VSMC phenotypic switching has led to the traditional binary classification of contractile and synthetic being reconsidered, since it is now clear that synthetic VSMCs can transdifferentiate into several different lineages, including foam cells, macrophage-like VSMCs, myofibroblast-like VSMCs and osteoblast-like VSMCs [reviewed in (53)]. The field has been aware that these synthetic subtypes exist (51), however, it is only with recent technologies, such as advanced lineage tracing and single cell RNA-Seq, that they have been explored in detail. Single cell sequencing analysis of aortic tissue indicated that the tunica media consists of five VSMC-like clusters, which are grouped according to their gene expression profiles and indicate pathways associated with contraction, stress responses, and cell cycling (with both low and high contractile protein expression), and also fibroblasts (54). The capacity of VSMCs to regulate these processes is well documented to be perturbed in many vascular diseases, particularly atherosclerosis [reviewed in (55)], but to date has not been well explored in terms of its involvement in arterial dissection pathophysiology. VSMC phenotype switching has been described as a pathological mechanism of aortic dissections (56), though importantly, this phenomenon is yet to be considered for other arterial dissection types, such as spontaneous coronary artery dissection (SCAD) or cervical artery dissection (CeAD). How synthetic VSMCs are activated in arterial dissections may differ between dissection subtypes in other vasculopathies and this knowledge is likely critical for understanding how and why these various vasculopathies occur.

Signaling from ECs is important for VSMC phenotype switching. VSMCs never exist *in vivo* without some proximity to ECs. Although separated by the internal elastic lamina, this layer offers little resistance to direct chemical communication between these cells as it is fenestrated, or discontinuous and fibrous (57). It has long been established that interactions between ECs and VSMCs affect cell morphology and proliferation of both these cell types (58). Studies of bovine aortic VSMC and EC co-cultured but separated by a permeable polyethylene terephthalate membrane, mimicking the internal elastic lamina, indicated a 56% increase in VSMC proliferation when VSMCs were co-cultured opposite ECs compared to VSMC-only cultures. Notably, the VSMCs co-cultured with ECs retained a spindle shape, whereas VSMC-only cultures were epithelioid in appearance (58), consistent with a contractile and synthetic phenotype, respectively (46). It is not clear how well these co-cultures reflect *in vivo* conditions where VSMCs vary in proximity to the ECs of the intima and vasa vasorum, however, 3D cultures of VSMCs and ECs may provide insights into their relationship in the context of phenotype modulation.

#### Anatomy and sex influence vascular smooth muscle cell identity

Like ECs, VSMCs vary based on anatomical location and between sexes. Reduced intimal-medial thickness, owing to a lower abundance of VSMC is observed in females (59, 60). Single cell RNA transcriptome analysis demonstrated sex-specific differences in the expression of collagen in murine aortic-derived VSMCs (54). More recently, a single cell study also highlighted that key drivers of atherosclerosis differ between sexes – unique VSMC phenotype-modulating mechanisms underpinning an increased risk of atherosclerotic lesion development in females (61). Collectively, these data highlight that like ECs, in both health and disease VSMC identity is highly nuanced with regards to the influence of sex.

### The extracellular matrix provides the structural qualities of the vasculature

It would be remiss to discuss the cells of the vasculature without also considering the most abundant feature of the arterial cell landscape – the ECM. A fascinating, dynamic environment, it is irrefutably a major player of the vasculature and is as important as the ECs and VSMCs themselves. The ECM accounts for up to 60% of the dry weight of a large vessel (2). Critical to the structure and function of the arterial wall, the ECM provides elasticity and distensibility, and acts to provide critical signals, both directly by interacting with adhesion molecules such as integrins, and indirectly, as a reservoir for signaling factors. ECM composition differs within the vessel wall, including that adjacent to ECs, in the lamellae formed by VSMCs, as well as at the interface between the contractile and non-contractile layers (intima-media and media-adventitia). Differences in ECM composition are critical for providing the specific mechanical and biochemical properties required to facilitate appropriate signaling for each unique microenvironment of the vessel wall, and for maintenance of arterial homeostasis (62).

There are two major macromolecules within the arterial ECM: elastic fibers and collagen fibers. Elastic fibers comprise a diverse range of ECM species, including elastin, fibrillin, microfibril-associated glycoprotein-1, latent TGF-β, decorin, biglycan, versican, microfibrillar-associated protein, tropoelastin, lysyl oxidase, fibulin, vitronectin, amyloid, collagens, and endostatin (63). Elastin is attributed with providing distensibility in the vessels and distributing stress onto collagen (64). Encoded by *ELN*, elastin is the major component of elastic fibers. Like collagen, elastin is also expressed variably in different anatomical locations (3). Decreases in elastin expression during development have been shown to result in thickening of lamellar units and increased abundance of VSMCs, which is thought to compensate for the reduced elasticity normally provided by the elastin in the ECM layer of the lamellae. Elastin insufficiency has been linked to hypertension in later life in both mice and humans (65).

Collagen fibers are comprised of bundles of collagen fibrils, which are formed from collagen triple helix bundles (each collagen triple helix being made up of three collagen chains). Collagen is the most abundant protein in mammals. There are many types of collagen chains – over 44 chains have been recognized (66). Different collagen types provide different mechanical properties, such as stiffness or elasticity (67). In arteries, collagen is the greatest facilitator of the contractile changes that occur and is attributed with defining the stiffness of vessels (64). The abundance of total collagen and collagen subtypes in the vasculature is not universal (67, 68), with differences in variables such as anatomy, sex, ethnicity and age still to be cataloged.

#### The extracellular matrix is essential to cellular identity

The ensemble of ECM proteins, known as the matrisome, encompasses 300 different proteins that provide the elegant and complex extracellular environment required to maintain cellular identity (66, 69). Both VSMCs and ECs contribute to the generation and modification of the arterial ECM, which is established during development, and has low turnover in adult life, except for alterations with aging, disease, and injury. For example, once established at birth, elastin turnover is thought to be only 1% per annum (70). Experiments as early as the 1980s showed that altering the ECM on which VMSCs are grown drastically changes the morphology of VSMCs, highlighting the importance of consistency in the ECM. Male rat aortic VSMCs change their phenotype, including their shape, attachment, and spread *in vitro*, with alterations in fibronectin, laminin, collagen IV or peptide coating (71). Even changes to the conformation of collagen can influence cellular phenotype: collagen 1 in its fibrillar form promotes a contractile VSMC phenotype, whereas monomeric collagen 1 activates VSMC proliferation, indicative of encouraging a synthetic phenotype (72). Elastin is similarly essential – in cultured primary porcine VSMCs, elastin reduces proliferation and migration in an inverse dose-dependent manner, while in ECs, proliferation was only reduced once elastin reached a threshold concentration (10 mg/mL) (73). Additionally, homozygous elastin knock-out-derived murine VSMCs express reduced levels of contractile myofilament-associated proteins, including ACTA2, CNN1 and TAGLN. The mechanisms driving this shift in phenotype have led to the finding that elastin can activate a G protein-coupled pathway leading to the inhibition of adenylate cyclase, which causes a reduction in cAMP levels and stimulates actin polymerization (74).

The mechanical properties of the arterial ECM modulate cellular phenotypes. Increased extracellular stiffness has been shown to influence an array of cellular properties, including focal adhesion expression and cytoskeletal structure (75, 76). Stiffness also influences expression of plasticity proteins. A study into the effects of altered substrate stiffness on mesenchymal stem cells (vascular progenitors) found that expression of the Yamanaka factors’ Nanog, Sox2, and Oct4, decreased with increased stiffness (77). This same study also found that reduced stiffness resulted in a more relaxed nucleus, leading to the speculation that this allowed for an increase in euchromatin that facilitated increased pluripotency gene expression (77). Interestingly, increased motility in the presence of a stiffer substrate, a response known as durotaxis, has been shown when VSMCs were plated on fibronectin, but not laminin, and an inverse relationship has been shown in a recent study comparing migration of VSMCs on collagen (migration decreased with increased stiffness) and fibronectin (again, migration increased with an increase in substrate stiffness) (78, 79). Together these studies suggest the effects of extracellular substrate stiffness rely not only on the mechanical properties of VSMCs but also on ECM composition.

The ECM provides a critical reservoir for growth factors and signaling compounds, such as TGF-β. TGF-β signaling is fundamental in mediating both EC and VSMC proliferation, cell death, migration and adhesion, cytoskeletal organization, as well as in regulating the ECM itself (80). This signaling is nuanced – eliciting differing effects depending on cell type, concentration, and receptor presence (81). In ECs, for example, TGF-β binding to the TGF-β receptor 2 (TGFβR2) and the ALK5 complex elicits downstream SMAD2/3 signaling, which inhibits proliferation and migration and, thereby, maintains quiescence. Conversely TGF-β interaction with the TGF-βR 2 and the ALK1 complex activates SMAD1/5 signaling, stimulating proliferation and migration (82). TGF-β is stored in a latent form in the ECM. Under normal physiological conditions, latent TGF-β is activated by cleavage of its propeptide (latency-associated protein) by furin. This activation occurs *via* a number of mechanisms, the primary being integrin activation (80).

#### Anatomy and sex influence arterial extracellular matrix

Given the complex variability in arterial cell identity, it is unsurprising that arterial ECMs vary beyond simple differences in abundances of collagen fibers and elastin fibers. The overall composition of the ECM varies in different anatomical regions – as aforementioned, expression of the ECM in ECs and VSMCs varies throughout the vascular tree. A microarray study of blood vessels harvested post-mortem indicated distinct differences in mRNA expression of ECM protein-encoding genes. Thus, ECM proteins, including collagen subtypes 5α1/2, 4α1 and 4α2, as well as many ECM interacting proteins, such as integrins, were found to be differentially expressed across anatomical regions, with a distinct partitioning when grouped by vessel size (83).

Given the differing functional requirements of the layers of the artery, vascular ECM must also vary in composition at the microenvironment level. The interfaces of the arterial layers must facilitate the coalescence of two differing cell types, which differ in function, and, therefore, structure. Endothelial ECM is characterized by great asymmetry in its composition – the luminal surface of ECs lacks the structural components of collagen and elastin fibers. Instead, the extracellular space is covered in a fragile mesh-like layer of polysaccharides, proteoglycans, glycoproteins and glycosaminoglycans, termed the glycocalyx, that appropriately translates to “sweet husk.” This layer, which appears almost as a fur lining, retains hyaluronan and heparan sulfate to create a hydrostatic pressure gradient (84). It plays an important role in preventing pathogen entry, and facilitating the diffusion of required nutrients (85). On the reverse, intramural side of ECs, the intimal-medial interface must facilitate the interactions of ECs with the contractile VSMCs of the media. Similarly, the ECM of the media, so integral to facilitating pulsatility, must coordinate the recurring units of VSMCs, yet it must also permit reticulation of vasa vasorum from the adventitia into the media.

Further to this complexity, artery composition and stiffness also varies based on sex and aging. Artery size varies with sex; average female arteries are smaller than their male counterparts (86, 87). Arterial stiffness increases with age. Rates of change are, however, sex-dependent such that arterial stiffening accelerates after the onset of menopause, reversing the trend in women, who, relative to men, have more compliant vessels (88, 89). This phenomenon is also observed in non-human primates (90). This relationship has been attributed to sex hormone levels such as estrogen and progesterone, which are lower in men, and which decrease in menopausal women. Progesterone and estrogen have been demonstrated to alter ECM deposition by VSMCs *in vitro*, thus reduced ECM regulation by these hormones is suspected to contribute to this increased arterial stiffness (91). However, a recent study of 339 women found that the arterial stiffness increase that occurred within the year following their final menses did not correlate with either estrogen or follicle stimulating hormone levels, though this study did not examine progesterone levels (92).

## Arterial cells and extracellular matrix dysfunction prime arteries for dissection

**FIGURE 4:**
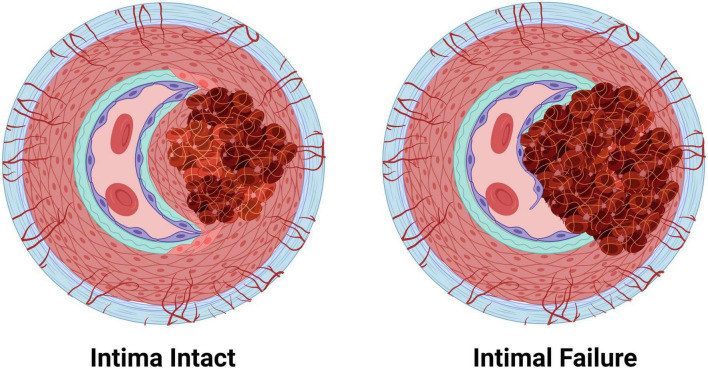
The two pathologies of arterial dissections: bleeding within the tunica media results in intramural hematoma formation with separation of the tunica media layer to create a false lumen that causes occlusion of the true lumen of the vessel, thereby preventing tissue perfusion. Some dissection events are associated with tunica intima tearing (intimal failure). Created with BioRender.com.

Arterial dissections occur when arterial structure is compromised, and, except for aortic dissections, development of an IMH prevents perfusion by the artery. Dissection falls into two categories (Figure 4): (1) an IMH forms within the media, while the intima remains intact, or (2) the integrity of the intimal layer is compromised resulting in the formation of a false lumen due to blood flowing into the medial layer of the artery from the true lumen, resulting in an IMH. It is not clear if this tear in the intima is a primary event or is secondary to expansion of an existing IMH, however, angiography, which requires the use of a thin guidewire for catheter placement has been known to cause an iatrogenic intimal tear and/or to exacerbate a spontaneous dissection.

Collectively, the localization of dissections within arteries implicates perturbations of ECs, VSMCs and/or arterial ECM in the etiology of arterial dissections. Schievink and colleagues, proposed in their 1994 review that primary arteriopathies are the result of single ECM protein mutations, the phenotypes of which are predicated on the distribution (and abundance) of ECM expression in different organs (93). Based on these considerations, it seems reasonable to hypothesize that the composition of different arteries is predicated on their mechanical requirements, and that dysfunction in critical ECM components at an anatomical position will predispose the vascular structure to dissection at such locations. It could also be speculated that the subtypes of dissection correlate with a specific Achille’s heel of arterial architecture i.e., that the various ECM compositions at different cellular interfaces of the artery (EC-EC, EC-VSMC, VSMC-VSMC, and EC-ECM layer, VSMC-ECM layer) serve as points of mechanical weakness when compromised by aberrant protein expression, with such weakness predisposing to dissection. Extending this notion, perturbations in EC and VSMC function, particularly VSMC contractility, may similarly compromise arterial integrity and contribute to susceptibility to dissection.

## Spontaneous arterial dissections

Spontaneous arterial dissections occur both in association with connective tissue diseases as well as independently, in otherwise seemingly healthy individuals. A commonly held hypothesis is that spontaneous arterial dissection events occur according to a two-hit model – requiring a genetic burden to predispose an individual to a dissection, and an environmental trigger to precipitate the event. Many of the pathways and genes that have been associated with spontaneous arterial dissections are shared amongst different dissection types, as discussed below.

Spontaneous arterial dissections may occur in many different anatomical locations, such as the cervical arteries and aorta, or there may be multiple dissections in one area, such as in multiple branches of the coronary artery (94). This strongly suggests that there are likely mechanistic subtypes of arterial dissection. Simultaneous artery dissections have been reported – one case study reported seven arterial dissections within 24 h of hospital admission in both iliac arteries, inferior mesenteric, renal, splenic and celiac arteries (95). Although no phenotypic features of connective tissue diseases were evident, genetic screening of the 28 vascular dissection and aneurysm-associated genes (a connective tissue disease panel) identified a variant in *COL3A1* (c.3199A > T, Ser1067Cys). The clinical significance of this variant is unknown; these amino acids differ only in one functional group whereby an alcohol in serine is replaced by a thiol group in cysteine (95). As there were other family members who were also homozygous for this variant with no history of vasculopathy, further investigation, such as whole genome sequencing (WGS) and cellular and molecular studies, is needed to elucidate the mechanisms causing this extreme phenotype.

Importantly, there are reports of family members carrying variable penetrance risk variants, who have no notable disease features and who have not suffered from a dissection (1, 96, 97). Whether there is a protective genetic element present in these individuals, or if the right positive environmental cues (or lack of negative environmental cues) are also present, is unclear. Future integration of WGS information to determine polygenic risk scores for dissection may be helpful for these individuals. The asymptomatic state yields hope that therapeutic intervention, to upregulate protective cellular processes, once defined, could prevent future dissections in family members at risk, and those at risk of recurrent dissections.

### Diseases affecting vascular extracellular matrix are associated with increased risk of arterial dissections

Increased risk of arterial dissections in a number of diseases that affect vascular integrity suggests a genetic predisposition to dissections. Patients with connective tissue syndromes including Ehlers–Danlos, Marfan syndrome, Loeys–Dietz, Alport, as well as those with osteogenesis imperfecta (brittle bone disease), polycystic kidney disease, and fibromuscular dysplasia (FMD), commonly report dissections in the aorta, coronaries, and/or cervical arteries. Pathogenic variants causing these conditions are overwhelmingly ECM-related (Table 1). Sexual dimorphism is reported in many of these conditions– for example in a study of vascular Ehlers–Danlos patients, men were more common in the aortic dissection cohorts, while women dominated in the SCAD and CeAD Ehlers-Danlos cohorts. While larger studies are needed to further validate these findings (98), this theme of sex-related differences is prominent among the different types of arterial dissection.

**TABLE 1:** Arterial dissection associated conditions.

Condition	Description	Genes with pathogenic variants	Prevalence	Sexual dimorphism	Dissection associated	References
Ehlers–Danlos (EDS)	CTD; hyperextendable joints, hyperextensible skin, easy bruising, abnormal scarring; vascular EDS subtype, and in 17% 13 non-vascular EDS have vascular involvement	*COL1A1, COL1A2, COL5A1, COL5A2, COL3A1, COL12A, ADAMTS2, PLOD1, FKBP14, TNXB, CHST14, DSE, B4GALT7, B3GALT6, SLC39A13, ZNF469, PRDM5, C1R, C1S, AEBP1*	1:5,000	Dissection types vary by gender	CeAD, aortic, SCAD	(98, 264–268)
Marfan syndrome	CTD; affects the ocular, skeletal, and cardiovascular systems with varying severity	*FBN1*	1:5,000–1:10,000	Sex related burden (pregnancy increases aortic root dilation)	CeAD, aortic, SCAD, PA	(109, 157, 220, 269–272)
Loeys-Dietz syndrome	CTD; affects the skin, skeletal and cardiovascular system	*TGFBR1, TGFBR2, SMAD3, TGFB2*	Less than 1:10,000	NA	CeAD, aortic, SCAD	(273–275)
Alport syndrome	Affects the renal, auditory and ocular systems. Hypertension increases risk of cardiovascular events 1000 fold.	*COL4A3, COL4A4, COL4A5*	1:10,000	X-linked in 85% cases	Aortic, SCAD	(276–279)
Fibromuscular dysplasia	Abnormal (dysplastic) cell growth in medium-sized arteries causing tortuosity	*PHACTR1*	Up to 6.6% population (potential kidney donors)	90% patients female; male patients significantly associated with CeAD	CeAD, SCAD	(157, 166, 171, 280, 281)
Polycystic kidney disease	Kidney cyst formation, cardiovascular	*PKD1, PKD2*	10M people globally	NA	CeAD, Aortic, SCAD, iliac	(34, 175, 282–284)
Osteogenesis imperfecta	Brittle bones disease	*COL1A1, COL1A2, BMP1, CRTAP, LEPRE1, PPIB, TMEM38B, SERPINH1, FKBP10, PLOD2, WNT1, CREB3L1*	1:20,000	NA	CeAD, aortic, SCAD	(229, 285–289)

### Aortic dissections

The aorta is the largest artery in the body, and the most common artery to dissect. It is subjected to the highest pressure of any artery, being the first to receive ejected, oxygenated blood from the heart. It is estimated that 1–2% of all deaths in the Western world are caused by weak and defective aortic structure (99). Thoracic aortic dissections are the most common catastrophic vascular structural failure, exceeding that of ruptured abdominal aortic aneurysms. Taking into account cases that are lethal prior to hospital admission, the annual prevalence of aortic dissections is 15 cases per 100,000 people (100).

#### Aortic dissection pathology

There are numerous clinical classification systems for aortic dissections. Traditional anatomical-based classification systems remain popular – namely the ternary DeBakey system (101) and the binary Stanford system (102), both of which classify dissections according to the extent of involvement of the ascending and/or descending aorta. Aortic dissections are also commonly classified by their anatomical locations into thoracic and abdominal regions. The majority (62.3%) of aortic dissections occur in the ascending portion of the aorta and are considered thoracic aortic dissections (103). Newer classification systems have been proposed, some of which consider features of pathology (e.g., where, within the vessel wall, the dissection forms; if the dissection was iatrogenic, and if atherosclerotic plaques were present) that provide useful information for understanding the underlying mechanisms. However, these have not yet been widely adopted (104, 105). It has been proposed that dissections occur at areas of the aorta subjected to the largest pressure changes; a notion supported by computational flow predictions (106). Vasa vasorum dysfunction is also increasingly recognized as being central in aortic dissection pathogenesis (107). This is strongly evidenced by a porcine study wherein occlusion of aortic vasa vasorum was sufficient to cause aortic dissection (108). Further investigations into vasa vasorum dysfunction are urgently needed.

#### Conditions and risk factors associated with aortic dissection

As the most common type of arterial dissection, aortic dissections are also the most well-defined in terms of associated risks. Sexual dimorphism is evident with males accounting for 67.5% of cases (109). Women who experience an aortic dissection tend to be older. Analysis of the International Registry of Acute Aortic Dissections revealed that of patients under 50 years old, males represented 80% of cases, however, this divergence steadily drops with age, reaching an almost 1:1 male:female ratio in patients over 75 years old (103). Notably, this study also showed dissection location partitioned with age – the mean age of patients suffering from an ascending aortic dissection was significantly younger than those who developed other aortic dissections.

Hypertension is a strongly linked risk factor for aortic dissections. In a Swedish study of thoracic and abdominal aortic dissections, hypertension was present in 86% of individuals (109). Smoking, which has been linked to hypertension, as well as general vascular damage, is also a high risk factor (110, 111). It is unclear whether this association is due to smoking causing hypertension, arterial damage, or both, and/or to another explanation entirely. Other agents that cause vascular injury have also been linked to aortic dissection including infections, such as syphilis (112). Though now rare, in cases of syphilis where the infection has been ongoing for more than 10 years, cardiovascular sequelae, including aortic dissection, are common, (113, 114). An increase in aortic dissections following COVID-19 infections has been reported and long term effects of infection are yet to be fully realized (115). Rare cases of aortic dissection have also been reported in people with ankylosing spondylitis, an inflammatory autoimmune condition that often causes aortitis (116).

#### Genetics and molecular mechanisms of aortic dissections

Perturbations in a number of pathways have been implicated in aortic dissections – TGF-β signaling, ECM, VSMC contractility, and VSMC metabolism (117). Importantly, these pathways are critical in cellular identity and phenotype switching in VSMCs, which we postulate precedes all forms of arterial dissection. The genetics of aortic dissections are subject to regular review. It is not the intent of this review to provide an exhaustive update, but rather a comparison for a holistic view of arterial dissections. By comparing findings with the growing wealth of knowledge provided by genetic and cellular studies, we can begin to elucidate the underlying disease mechanisms shared by all arterial dissections. Moreover, contrasting the differences in these dissection subtypes can direct our understanding of the susceptibility of specific anatomical locations to dissection.

##### Transforming growth factor β signaling dysfunction

Given the well-established link between TGF-β signaling dysfunction and disease-associated aortic dissections, such as in Marfan Syndrome, it is unsurprising that several members of this pathway have been implicated in spontaneous aortic dissections as well as connective tissue diseases. Variants that have been associated with thoracic aortic dissection (through syndromic affiliations, such as Loeys-Dietz) include two of the three isoforms of TGF-β (*TGFB2-3*), both TGF-β receptors, *TGFBR1* and *TGFBR2*, and TGF-β-latent transforming growth factor β-binding protein 1 and 3 (*LTBP1, LTBP3*) (118–120). Protein-protein interaction analysis has implicated TGF-β1 as a central protein in aortic dissections (121), though variants in *TGFB1, per se*, have not been identified in patients.

The downstream effectors of TGF-β signaling, such as the SMAD family, have also been linked to aortic dissections. *SMAD3* is regarded as a definitive causal gene for thoracic aortic dissections (122). SMAD2, SMAD4 and SMAD6, which signal through c-Jun/c-Fos (123), are also associated with aortic dissections (1, 118, 120, 124, 125). *ZFYVE9*, encoding zinc finger FYVE domain-containing protein 9, which recruits SMAD proteins and is involved in TGF-β signaling, has been associated with aortic dissections through recent whole exome sequencing studies (121). Similarly, LRP1 has recently been associated with acute aortic dissections (126). Loss of LRP1 recapitulates Marfan syndrome disease mechanisms, wherein TGF-β is prematurely released from the ECM. Interestingly, loss of LRP1 upregulates the JNK1/2-c-Jun-Fra-2 signaling pathway in myofibroblasts (127), a pathway also affected in aortic dissections.

Mutant forkhead box E3 (*FOXE3*) has been identified to predispose to aortic dissections (128). Not a lot is known about the role of FOXE3, a transcription factor better known for its critical role in lens epithelial cell proliferation and survival. In mouse lens epithelial cells, forced persistent expression of *FOXE3* during development alters the cytoskeleton and the ECM, and causes prolonged upregulation of TGF-β3 and CTGF expression (129). *FOXE3*-deficient mice have increased VSMC apoptosis, as well as increased aortic pressure, and rupture (128). Methionine adenosyltransferase 2A (*MAT2A*) (130), another aortic dissection risk-associated gene, has previously been suggested to be a VSMC metabolism gene, however, recent work in hepatic stellate cells has also identified MAT2A as a downstream target of TGF-β. Upregulation of TGF-β1 increased MAT2A concentration *via* p65 phosphorylation, with subsequent increased expression of both ACTA2 and COL1A1 (131).

##### Extracellular matrix dysfunction in aortic dissections

Variants in an array of collagen subtypes are associated with aortic dissections, including eleven collagen genes: (*COL1A1, COL1A2, COL3A1, COL4A1, COL4A5, COL5A1, COL5A2, COL9A1, COL9A2, COL11A1*, and *COL18A1*) (1, 118, 120, 121, 124, 125, 132, 133). Variants in a range of quintessential ECM proteins are also involved, including EGF-containing fibulin-like extracellular matrix protein 2 (*EFEMP2*), microfibril associated protein 5 (*MFAP5*), lysyl oxidase (*LOX*), elastin (*ELN*), and fibrillin (*FBN*)-1 and -2 (121, 134, 135).

Dysregulation of the ECM is a regularly occurring theme in aortic dissection-associated variants. Downregulation of the hsa-miR-29 family, which has been shown to increase collagen levels, including in cardiac fibrosis where it has been shown to increase collagen1A1, 1A2, 3A, and fibrillin-1 mRNA expression in regions affected by myocardial infarction in mice (136), was detected in aortic tissue of dissection patients compared to healthy controls (132). A decrease in HDAC6 protein levels has also been detected in aortic tissue of dissection patients compared to coronary artery disease patients, whereas mRNA levels of ECM proteins were found to be increased, including COL3A1 and COL1A2, matrix metalloprotease 2 (MMP2), tissue inhibitor of metalloproteinases 2 (TIMP2), periostin (POSTN) and connective tissue growth factor (CTGF). This ECM regulation of matrix secretion is thought to involve HDAC6 deacetylation of H3K23 (137). MMP1 (total) and MMP9 (total and active) levels have been shown to be increased in aortic dissection tissues compared to controls (138).

Suppressor of cytokine signaling 3 (*SOCS3*) is a more recent gene to be implicated in the pathogenesis of aortic dissections. In a mouse model of aortic dissection induced by minipump administration of the lysyl oxidase inhibitor, β-aminopropionitrile (BAPN, 150 mg/kg/day), and the vasoconstrictor, angiotensin II (1,000 ng/kg/min), it was found that knockout of SOC3, an activator of JAK/STAT and negative regulator of Janus kinases/signal transducer, led to a decrease in aortic dissections (139). Aortae from SOC3 knockouts had increased tensile strength, likely due to an increased deposition of total collagen, which was significantly higher in the adventitia. This suggests that increased tensile strength reduces the incidence of dissections (139). Additionally, levels of several ECM-associated proteins have been found to be decreased in aortic dissection tissue, including HSP27, SOD3 and osteoglycin (140).

##### Cytoskeletal dysfunction in aortic dissection

Proteins of the cytoskeleton, as well as cell adhesion-associated proteins that anchor vascular cells to the vascular ECM, feature heavily in the proteins that are associated with aortic dissection. Altered expression of VSMC intracellular contractile proteins, including MYH11, myosin light-chain kinase (encoded by *MYLK*) that phosphorylates myosin light chain, ACTA2, and TAGLN have been associated with aortic dissections. In addition, *ACTA2* mRNA has been found to be increased in aortic tissue of dissection patients (137), suggestive of cells undergoing phenotypic changes. An early proteomics study also implicated an upregulation of ACTA2 protein with concomitant decrease in TAGLN protein in aortic tissues from patients post-dissection compared to controls (140). Talin-1, which tethers the ECM to the cytoskeleton by tethering actin to integrin, and is involved in the regulation of focal adhesions, integrin signaling, proliferation and migration, has been shown to be downregulated in aortic dissection tissue compared to normal controls (141, 142). Variants in the cell adhesion neurogenic locus notch homolog protein 1 gene, *NOTCH1*, are associated with aortic dissection/aneurysm (143). Moreover, protein levels of NOTCH1 have been shown to be reduced in thoracic aortic dissection tissue, despite an upregulation of *NOTCH1* mRNA (144). Collectively, the altered expression of these cytoskeletal proteins is suggestive of cellular activation and remodeling.

Dysfunction in the regulation of cytoskeleton proteins has also been associated with aortic dissections. A zinc finger protein, four and a half LIM protein 1 (encoded by *FHL1*), which regulates the structure and formation of myosin filaments (122, 145), was found *via* proteomics to be decreased in aortic tissue (140). This has also been independently corroborated in Western blot analyses of aortic tissue from dissection sufferers, which found a 2.5-fold decrease in FHL1 level in patients compared to controls. Importantly, immunohistochemistry revealed this decrease was most pronounced in the area of the media surrounding the tear when compared to the intima and adventitia (122). In the myoblast C2C12 cell line, FHL1 has been observed to potentiate the effects of TGF-β (146). In non-diseased vessels, FHL1 has been observed to increase with increased blood pressure (147), and has been shown to be increased in rat VSMCs following treatment with hypertrophic stimuli (148). siRNA knockdown of FHL1 in rat VSMCs caused a decrease in cell proliferation, which was not associated with apoptosis (122). Together these data suggest that FHL1 plays a role in VSMC plasticity (contractility/synthetic identity), though the exact role of this protein in VSMC phenotype regulation, and its role in vascular dissection will require further investigation. Similarly, variants of Unc-51-like kinase 4 (*ULK4*), a pseudokinase thought to remodel the cytoskeleton, have been linked to aortic dissections (126), with variants in *ULK4* having also been linked to hypertension (149, 150).

##### Metabolic dysfunction in aortic dissection

Metabolic changes are reported to precede aortic dissections, and a number of genes associated with aortic dissections are linked to metabolism (125). *DAB2IP*, encoding disabled homolog 2-interacting protein, which is involved in cell growth and survival and has been associated with aortic dissection as well as abdominal aortic aneurysms (121, 151). mRNA expression of both *CREBBP*, (encoding CREB-binding protein) and *EP300* (encoding histone acetyltransferase p300), both of which regulate cAMP-associated genes, was found to be downregulated in patient aortic dissection tissue compared to healthy controls (121). Interestingly, the aforementioned study by Liao et al. (140) focused on the role of oxidative stress as a mitigating pathway for aortic dissection (140), which is further supported by the recent identification of mitochondrial dysfunction being modulated by ECM stiffness in Marfan syndrome-associated aortic aneurysm formation (152).

Variants in the glucose transport 10 protein, Glut10 (encoded by *SLC2A10*), have been associated with a Marfan syndrome-like disease pathology that is seen in arterial tortuosity syndrome, another disease associated with aortic dissections. (100, 153). Variants in *SLC2A10* lead to a decreased density of Glut10 transporters, which causes ECM disarray and subsequent inappropriate TGF-β signaling, as well as oxidative stress (154). Notably, pathogenic variants in glut10 have been shown to alter angiogenesis (155). *CBS* encoding cystathionine-beta-synthase, has been categorized as a low-risk gene for aortic dissection (120). Variants in *CBS* are associated with the metabolic condition, homocystinuria, which, similar to pathogenic *SLC2A10* variants, can result in a Marfan syndrome-like disease pathology. Conversely, Group V secreted phospholipase A2 (sPLA_2_-V) is believed to play a protective role against aortic dissection. Increasing downstream mobilization of sPLA_2_-V substrates has been shown to rescue an angiotensin II infused murine model of aortic dissection. Thus, increasing dietary oleic acid and linoleic acid, which are normally mobilized by sPLA_2_-V, eliminated spontaneous dissection observed in 45% of sPLA_2_ knockout mice (*Pla2g5^–/–^*) (156).

### Spontaneous coronary artery dissection

The coronary artery, which supplies blood to the heart itself, is the only artery to perfuse its cognate tissue, the myocardium, during the relaxation phase of the cardiac cycle (diastole) rather than in systole. As with other muscular vessels, the outer layer of the coronary tunica media is perfused by vasa vasorum that originate from sites of branching of the coronary vessels (9) with little if any contribution from vasa interna (8). Dissection of the coronary artery results in an acute coronary syndrome (myocardial infarction or unstable angina) or death (157). As with all dissections, advances in their detection, *via* CT or MR angiography and direct intravascular imaging modalities (intravascular ultrasound and optical coherence tomography), have increased the diagnosis of spontaneous coronary artery dissections (SCAD) that were previously likely to be markedly underdiagnosed. It is now estimated that up to 4% of acute coronary syndromes are caused by a coronary artery dissection (158).

#### Spontaneous coronary artery dissection pathology

Most dissections in the coronary artery (∼70%) do not present with an intimal flap, indicating that IMH formation from vasa vasorum rupture is likely central to SCAD pathophysiology. There is a correlation between areas of the coronary artery that are more susceptible to dissection and a lower vasa vasorum density; the left anterior descending coronary artery (60% of dissection cases) has a density of 1.2 × 10^–5^ vasa vasorum/μm^2^, compared to the left circumflex (38% of dissection cases), which has 1.88 × 10^–5^ vasa vasorum/μm^2^, and the right coronary artery, (7% of dissection cases) that has a density of 2.14 × 10^–5^ vasa vasorum/μm^2^ (157, 159). One study has reported increased vascularization in the adventitia of post-mortem sections following fatal dissections compared to nonobstructive coronary artery disease patient sections (160). While it is unclear if this preceded or was a consequence of the SCAD, increased vascularization of this outermost layer may be due to a localized dysfunction, such as an increase in ischaemic areas within the media, which would promote angiogenesis in vasa vasorum (9).

#### Spontaneous coronary artery dissection associated conditions and risk activities

There is a distinct sexual dimorphism in SCAD incidence with 82–98% of cases occurring in women aged between 45 and 52 years (157, 161, 162). An estimated one third of myocardial infarctions in women under 50 are caused by SCAD. Risk of recurrence of SCAD has been reported to be up to 30% (163), although a recent prospective observational study only reported recurrence in 2% of cases over a median follow-up of over 2 years (164). The risk factors associated with SCAD are similar to those for aortic dissections. Hypertension and migraine are found commonly in SCAD survivors, being present in 45 and 43% of cases, respectively (94, 161). FMD is common in SCAD patients. SCAD and FMD share many of the same risk loci; FMD being reported in 45–86% of cases (165, 166). Such marked overlap suggests SCAD may be a manifestation of FMD specific to the coronaries, although it is intriguing that the classical vessel beading of FMD is not observed in the coronary arteries of SCAD patients (167). Inflammatory disorders and infections are rarely associated with SCAD. For example, like aortic dissections, there is anecdotal evidence that SCAD can occur in association with tertiary syphilis (168, 169). In addition, SCAD has been reported in a recently recovered COVID patient (negative nasal swab, positive for COVID-19 IgG antibodies) (170).

#### Spontaneous coronary artery dissection genetics and molecular mechanisms

Though little research has been done investigating the molecular mechanisms of SCAD, a number of genomic studies have been undertaken (97, 163, 171, 172), which indicate that genetic risk for SCAD can result from variants in multiple genes. The common nature of these variants suggests SCAD is likely a polygenic disease and requires an additional environmental stress for a dissection to occur. A meta-analysis of case control studies identified the first risk locus as the A-allele of rs9349379, a single nucleotide polymorphism located in an intron in the *PHACTR1/EDN* gene, which was found with a frequency of 0.72 in SCAD cases compared to 0.56 in controls (OR 1.67; *p* < 6.67 × 10^–21^). Identification of this locus sparked debate over the level of endothelial involvement in SCAD as the rs9340379 locus is located in an intronic region of *PHACTR1*, which is also a putative enhancer of the upstream gene encoding endothelin 1 (*EDN1*) (171), an endothelially expressed potent vasoconstrictor peptide. A clinical study has attributed deficient EC function to a difference in vascular function observed in SCAD patients, however, it failed to consider the role of VSMCs in the decreased peripheral arterial tone (vasoconstriction/dilation) observed in SCAD patients (173). Conversely, coronary blood flow studies suggest that endothelial dysfunction is not a principal cause of coronary artery dissection (174). Given the involvement of VSMCs in other vascular dissection disorders, it is unlikely that deficits in endothelial function alone would cause all SCADs, however, cellular studies will be critical in understanding how these two cell types contribute to the pathophysiology of coronary dissections. More recently, several other potential genetic risk loci have been identified as a result of WGS studies and genome wide association studies (GWAS), as detailed as follows.

##### Transforming growth factor β signaling dysfunction in spontaneous coronary artery dissection

Proteins directly and indirectly involved in the TGF-β signaling pathway have been strongly implicated in SCAD pathophysiology. Recently, a targeted and genome-wide analysis of a cohort of 91 SCAD patients revealed an enrichment of TGF-β when rare variant collapsing analysis was performed (172). Variants in *TGFB2* and *SMAD3*, as well as *PKD1* have been associated with SCAD in WGS analyses (175). Additionally, variants in *FBN1* have been associated with SCAD in a GWAS, as well as from WGS analysis, which identified two likely pathogenic variants (97, 172). Fibrillin has also been proposed as a SCAD biomarker, being elevated in SCAD patients compared to other acute coronary syndrome patients and healthy controls (176). However, fibrillin 1 is not specific for SCAD as it has also been found to be elevated in other vasculopathies including aortic and cervical dissections (177).

Variants in SCAD-associated genes, *TBX2*, a T-box transcription factor; *YY1AP1*, yin yang 1 (YY1)-associated protein 1; *F11R*, encoding junctional adhesion molecule-A (JAM-A), and *LRP1*, which is also associated with aortic dissections, have been shown to affect cellular proliferation through pathways downstream of TGF-β (175, 178–182). *GLI3*, which was identified as a gene linked to SCAD in the Carss et al. (175) patient cohort, has also been thought to be linked to TGF-β signaling. Encoding a transcription factor in the sonic hedgehog pathway, *GLI3* is suggested to be a repressor of TGF-β-dependent hedgehog pathway activation, which is critical for proliferation and cellular identity (183, 184).

##### Extracellular matrix dysfunction in spontaneous coronary artery dissection

Similar to aortic dissections, collagen variants have been associated with SCAD based on a recent whole exome sequencing study (185). Variants of a rare and disruptive nature (being pathogenic or likely pathogenic) were found to be enriched in a cohort of 130 SCAD patients in the ECM structural constituent conferring tensile strength pathway (GO:00300200), which included *COL1A1, COL1A2, COL3A1, COL4A1, COL5A1, COL5A2, COL6A1, COL12A1* and *COL27A1.* Variants in *COL3A1* and *COL4A1* were also identified by Tarr et al. (172). Carss et al. (175) highlighted variants in other genes, including *COL18A1* and *COL4A2*, and SRY-Box transcription factor 9 encoded by *SOX9* (186), as having possible associations with SCAD, however, they did not reach their required significance thresholds, but scored highly and were plausibly associated with SCAD. This same study also identified that variants in the gene for the protein transport protein, *SEC24B*, were SCAD-associated, with expression of *SEC24B* having since been found to be involved in collagen export (187).

A number of ECM glycoprotein genes have also been associated with SCAD *via* GWAS studies, including *ECM1* and *ADAMTSL4* (97, 163, 188). ECM1 is associated with cellular migration and cellular phenotype transition, and has been shown to inhibit activation of TGF-β (189, 190). ADAMTSL4 binds with fibrillin-1, and accelerates the biogenesis of microfibrils (191). Turley and coworkers also suggested that a secreted protein, encoded by *C1orf54*, is associated with SCAD; C1orf54 has previously been linked to carotid artery aneurysm, although the function of this protein is still unknown (97).

*FMR1* has been linked to SCAD. Premutations in this gene, which encodes fragile X mental retardation protein (FMRP), are thought to increase susceptibility to dissections, since FMRP is involved in regulating the ECM and cytoskeleton (192). Impaired cytoskeletal protein-function has been shown in embryonic fibroblasts of *FMRP* knockout mice (193), and increased plasma levels of MMP9 are observed in Fragile X patients as compared to healthy controls (194). Of note, hypertension is also common in patients with Fragile X syndrome, though the mechanism for this is unclear (195).

SCAD studies have also identified pathogenic variants of *ABCC6* (or loci associated with this gene) in patients (172, 188). Encoding ATP Binding Cassette Subfamily C Member 6, variants in this gene cause *pseudoxanthoma elasticum*, a connective tissue disorder that affects vision, skin and the cardiovascular system to varying degrees (196, 197); cardiovascular sequelae occurring later than cutaneous and ophthalmological manifestations (198). It is thought that an increased degradation of elastin, as well as the presence of calcium/phosphorus deposits contribute to this arterial pathology (199). Transmission electron microscopy imaging of the dermis of *pseudoxanthoma elasticum* patients showed irregular and clumped elastic fibers, as well as disorganized collagen, with some studies finding that collagen fibers are also misaligned (200, 201). Carotid arteries of these patients have been shown to have an increased intima/media thickness, and peripheral arteries (intracranial carotid, and arteries of the limbs) show precocious calcification (202).

##### Cytoskeletal dysfunction in spontaneous coronary artery dissection

Similar to aortic dissections, pathogenic variants in the talin 1 gene, *TLN1*, have been identified in a SCAD family cohort, along with 10 sporadic cases (203). Recent molecular studies have linked PHACTR1, LRP1, fibrillin 1, and talin 1 through indirect association; these proteins all commonly interact with integrins, acting to bridge the cell and the ECM (97, 163). Variants in cytoskeletal proteins including myosin light-chain kinase (encoded by MYLK), which phosphorylates myosin light chain, MYH11, and MYLK2, have also been linked to SCAD (172, 175). *NFATC1*, which encodes nuclear factor of activated T cells c1, is a transcription factor downstream of LRP1; its signaling regulates the extracellular bone morphogenetic protein–binding endothelial regulator pathway (204), and has been associated with modulation of VSMC cellular identity (205).

*HDAC9*, encoding histone deacetylase 9, was implicated in SCAD as a highly ranked gene in collapsing analysis by Carss and colleagues (175). HDAC9 is associated with prevention of calcification in the media whereby *HDAC9* knockdown has been shown to increase calcification, and its overexpression *via* adenovirus reduced calcification in cultured murine VSMCs as well as in a mouse calcification model of high phosphate treatment (206). An *HDAC9* variant is also associated with large vessel stroke (207). Importantly, HDAC9 has been shown to repress contractile protein gene expression in murine aortic tissue (208).

##### Metabolic dysfunction in spontaneous coronary artery dissection

A likely pathogenic variant in *ALDH18A1*, which encodes aldehyde dehydrogenase 18 family member A1, also known as pyrroline-5-carboxylate synthetase (P5CS) (209), was identified in a cohort of 91 SCAD cases (172). Variants in *ALDH18A1* have been reported to cause *cutis laxa*, a rare connective tissue disorder associated with abnormal ECM. *Cutis laxa* is also linked to the aortic dissection-associated genes, *EFEMP2* (fibulin 4), *SLC2A10, ELN*, and the related genes, *FBLN5, LTBP4* (210). A variant in *ALDH18A1* has been found to reduce cellular arginine, and dietary arginine supplementation of a *cutis laxa* patient carrying this variant has been shown to attenuate disease symptoms. Importantly in the context of SCAD, arginine is critical for both ECM and NO synthesis (209).

A variant in *TSR1*, a ribosome maturation factor, was identified in a predominantly male Chinese Han SCAD cohort (211). However, patients with atherosclerosis were not excluded from this study so the implications of this finding are not yet clear, and they have yet to be replicated (212). In support of this finding, however, is the identification that variants in other protein synthesizing genes have been associated with SCAD, including mitochondrial ribosomal protein S21 (*MRPS21*) and peptidyl-glycine alpha-amidating monooxygenase (*PAM*) (175). Variants in two VSMC proliferation genes, *ARNTL* and *LINC00310*, have also been associated with SCAD. These genes encode the circadian clock regulating transcription factor, aryl hydrocarbon receptor nuclear translocator-like protein 1, and a long non-coding RNA, respectively (97, 175, 213, 214).

### Cervical artery dissections

Cervical artery dissections (CeADs), encompassing dissections of the carotid and vertebral arteries, can present as migraine, or can cause a stroke, which may be fatal (215). Improvements in non-invasive imaging have led to increased reporting of these dissections in stroke patients. CeADs are believed to be the cause of up to 25% of strokes in young and middle-aged adults (under 50) (216). The prevalence of these dissections is now estimated to be at least 5 cases per 100,000 individuals (217), and more than half of CeAD patients will develop a stroke (218).

#### Cervical artery dissection pathology

As with aortic dissections and SCAD, CeADs present with an IMH, that may or may not be associated with an intimal flap, with pseudoaneurysms also being found. In some cases that involve an intimal tear, the endothelium is described as being irregular (218). Interestingly, in an etiological investigation into connective tissue disorders in 65 cervical artery dissection patients, 55% displayed ultrastructural aberrations in collagen, similar to those seen in Ehlers-Danlos syndrome, with only 5% of the patients having clinical manifestations of skin, joint or skeletal abnormalities. These findings strongly implicate vascular-specific ECM defects as an important component of disease etiology (219). This notion is reinforced by reports of cystic medial necrosis/mucopolysaccharide accumulation in the tissue of patients (220), and the recent identification of an ECM signature in recurrent CeAD patient skin biopsies detected by proteomics analysis (221). A post-mortem histology study performed on the superficial temporal artery of CeAD patients described an increase in vasa vasorum density, as well as the presence of micro-hematomas in CeAD patients compared to cadaver controls (222), although it remains unclear if these findings precede or are merely secondary to the dissection.

#### Cervical artery dissection associated conditions and risk factors

In a study of CeAD patients, sexual dimorphism was evident with 57% patients in the study being male. Interestingly, females who accounted for the remaining 43% were significantly younger with an average age of 42.5 years, compared to 47.5 years for male patients (223). It is unclear to what extent referral bias contributed to this divergence in patient populations, however, this follows the same trend of age-based gender risk observed in aortic dissection and SCAD (109, 161). A history of a cerebral aneurysm has been reported in almost 1 in 5 CeAD patients (18.2%), suggesting a common genetic risk (224).

Hypertension (225) and migraine are known risk factors for CeAD (226, 227). Acute infection increases the risk of CeAD, with acute infection 1 month prior to CeAD having been reported in nearly one third (31.9%) of cases, compared to 13.5% of controls (228). The frequency of infection was lower in those with a single artery dissection (odds ratio, 2.1) than in those with multiple arteries affected (odds ratio, 6.4) (228). As with aortic dissections and SCAD, again there are case studies linking syphilis infection with CeAD (113, 114). Developmental defects in the neural crest, resulting in congenital heart defects, are thought to link heart development with CeAD (229).

#### Cervical artery dissection genetics and molecular mechanisms

The genetic risk factors for CeAD are less well studied compared to aortic dissection and SCAD, with few WGS and GWAS studies being reported to date. Nonetheless, gene variants that increase the risk of CeAD follow a similar theme to those of aortic dissection and SCAD, including associations with TGF-β signaling, ECM and cytoskeletal protein genes, and metabolism associated genes. Variants in *TGFBR2* (230, 231), dual specificity protein phosphatase 22 (*DUSP22*), (232), *LRP1* and *PHACTR1* have all been associated with an increased risk of CeAD (233). Variants in ECM protein genes associated with CeAD include those for *COL3A1, COL4A1*, and *COL5A2* and *FBN1* (230, 232, 234, 235). Proteomic analysis of skin punch biopsies from patients with recurrent CeAD implicates ECM proteins whereby comparison of the proteomes of six recurrent CeAD patients with those 12 healthy controls that detected increases in perlecan, laminin-β2 (encoded by *HSPG2* and *LAMB2*, respectively) and COL12A1, and decreases were detected in COL1A2, COL4A2 as well as ELN and microfibril associated protein 5 (*MFAP5*), respectively. Western blot analysis of these biopsy samples also revealed a decrease in COL1A1 (221).

Variants in the genes for both intercellular adhesion molecule 1 (*ICAM1*) and cytoskeletal alpha-1-syntrophin (*SNTA1*) have also been associated with CeAD (232, 236), whereas associations between CeAD and metabolism involve variants in methylenetetrahydrofolate reductase (*MTHFR*), which regulates homocysteine levels (231), and alterations in homocysteine are associated with thrombosis and atherosclerosis that occur with deficiencies in folate, vitamin B_6_ and vitamin B_12_ (237). This is of interest as methionine adenosyltransferase 1A is an aortic dissection-associated protein and is also modulated by folate; low plasma folate levels were found to increase the risk of cervical artery dissection in a cohort of 39 patients (238).

### Rarer arterial dissections

#### Pulmonary artery dissections

The pulmonary artery is responsible for perfusion of the lungs and, uniquely, is the only artery to carry deoxygenated blood. A recent comprehensive literature review found only 150 reported cases of pulmonary artery (PA) dissections (239). As with many of the dissection disorders, detection of PA dissections has increased exponentially since the first identification described post-mortem in 1842 (239, 240), presumably due to better diagnostic technologies. Patients diagnosed antemortem have a survival rate of 70.5% – a stark contrast to early reports, which deemed the condition almost certainly fatal. Increased reporting correlated with increased antemortem reporting (239), suggesting the condition has been previously underreported due to a surprisingly high survival rate coupled with a low detection rate and that PA dissections are not as ultra-rare as once thought.

**FIGURE 5:**
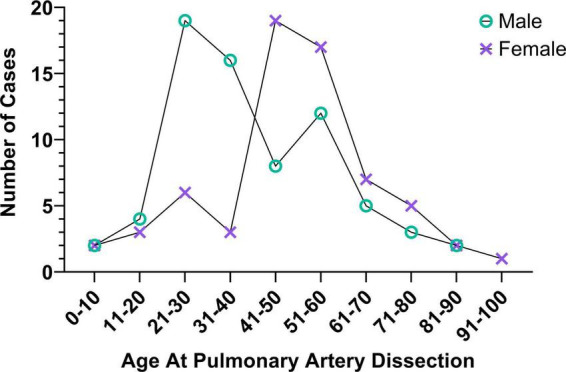
Risk profiles of pulmonary artery dissection patients. Like sufferers of aortic dissections, SCAD, and CeAD, pulmonary artery dissection occurrence varies with age and sex; data from (239).

Like other dissection events, PA dissections occur most commonly in the young to middle aged with three quarters of PA dissections patients being 21–60 years (239). Sexual dimorphism is less apparent in PA dissections, with a slight tendency to occur more frequently in males than females (ratio 1.1:1). However, as with the aforementioned arterial dissection types, cases diverge with both age and gender (109, 161, 223) where males represented 76% of cases in the age group 21–30, while females represented 70% of cases in the age group 41–50 (Figure 5).

Although the pathology of PA dissections is not well studied, dissections occur both with and without an intimal tear (240–242). In addition, post-mortem pathology of one patient indicated accumulation of acid mucopolysaccharides in the media, and degeneration and disorientation of the elastic medial layer (240); mucopolysaccharide deposition in the media having also been reported in a pregnancy-associated rupture of a dissecting aneurysm (243). These findings suggest that PA dissections and dissecting aneurysms may result from cystic medial necrosis and, in this regard, it is of interest that the pulmonary artery is richly endowed with medial vasa vasorum, which are required for perfusion and integrity of the medial layer. It is also noted that PA dissections are difficult to discern from dissecting pulmonary aneurysms (244–246).

Pulmonary hypertension is the strongest linked risk factor for PA dissections, with an estimated 50% of patients suffering from pulmonary hypertension and, independently, 9.8% from systemic hypertension (239, 242). Following hypertension, the next most common risk factor, yet far less mentioned in case studies, is a strong link to congenital heart disease; such abnormalities also predisposing to pulmonary aneurysms (229, 245, 247). To date, no genetic studies have been performed for PA dissections.

#### Renal and visceral artery dissections

Primary dissections of renal and visceral arteries (including the splenic, hepatic and mesenteric) are relatively uncommon, but they may occur secondary to extension of thoracic aortic dissections. It is possible, therefore, that these dissections represent extreme phenotypes, which may be instructive. Importantly, understanding the mechanisms underlying these rarer forms of dissection may provide insights into the pathogenesis of these diseases. Renal dissections are the second most common cause of renal infarct after embolism (248). They are classified into three groups based on cause (1) iatrogenic, (2) agonal (associated with renal failure, cirrhosis, and/or sepsis), or (3) spontaneous. Asymptomatic visceral dissections often produce few or no symptoms (249), suggesting that similar to SCAD and CeAD, these dissections are underdiagnosed.

Diagnosis of renal and visceral artery dissections typically only occurs if a computed tomography scan is performed as traditional imaging methods are not sensitive enough to detect a dissection, again suggesting that this is an under-diagnosed condition (250). This difficulty in detecting renal and visceral dissection may also explain why the first case of renal artery dissection was not reported until 1944 (251). Moreover, a quarter of renal artery dissections are only diagnosed at autopsy (250). As with other dissections, detection requires visualization of either an intimal flap and/or an intramural hematoma or both (252, 253).

Renal artery dissections predominantly occur in men at a 10:1 male to female ratio (250). Likewise, mesenteric and splenic artery dissections occur much more commonly in men (>80% of cases) (254, 255). Hypertension is linked to renal dissections and visceral artery dissections (256). Unlike many other types of dissections, the presence of atherosclerosis is not considered a basis to exclude spontaneous arterial dissection in renal or visceral dissection diagnosis (257, 258). Similar to PA dissections, no genetic studies have been performed for renal or visceral artery dissections.

## Discussion – Common features of dissections

By considering arterial dissections as a collective, we gain insight into both the commonalities and unique features of this devastating family of vascular disorders (Table 1). Despite differing consequences of arterial dissections – from myocardial infarction to stroke – the pathological presentation of dissections is consistent: arterial dissections present as an IMH (with or without an intimal flap). Common risk factors for arterial dissection patients include hypertension, tortuous vessels, and/or a history of vascular infection. Currently, there are no specific preventative therapeutics for arterial dissections, however, patients are often prescribed therapies such as the angiotensin II blocker, losartan, not only to manage hypertension (113, 114, 153, 228), but also because of its unique reverse-remodeling properties, which other antihypertensive agents such as angiotensin converting enzyme inhibitors lack. In Marfan syndrome, for example, the pathological changes in the aortic root are thought to be related to angiotensin II receptor 1 (ART1) signaling and the reverse-remodeling effects of losartan are mediated by blocking ART1 signaling and downstream TGF-β signaling (259). Targeted delivery of TGF-β inhibitory peptides has also recently been suggested as a potential future therapeutic for SCAD (260). Importantly, these stimuli are known to alter cellular identity, increasing cellular activation in ECs and VSMCs, and to alter the ECM (36, 46). The risk of arterial dissections is influenced by both sex and age, being more common in one gender than the other at different ages. This age-gender risk also varies depending on the anatomical location of the arterial bed involved. Vascular aging and sex contribute to differences in the arteries of men and women – particularly differences in stiffness and distensibility (90), but even at the molecular cellular level, in ECs, VSMCs, and the ECM. These differences undoubtedly contribute to differences in risk-demographics for various types of arterial dissections. β-blocking drugs, particularly the β1-adrenergic receptor blocker, metoprolol, are commonly used in the management of arterial dissections, both as anti-hypertensive agents and to reduce vessel shear stress. Moreover, in an observational study, β-blocking drugs (metoprolol or bisoprolol) have been shown to reduce SCAD recurrences (261).

**FIGURE 6:**
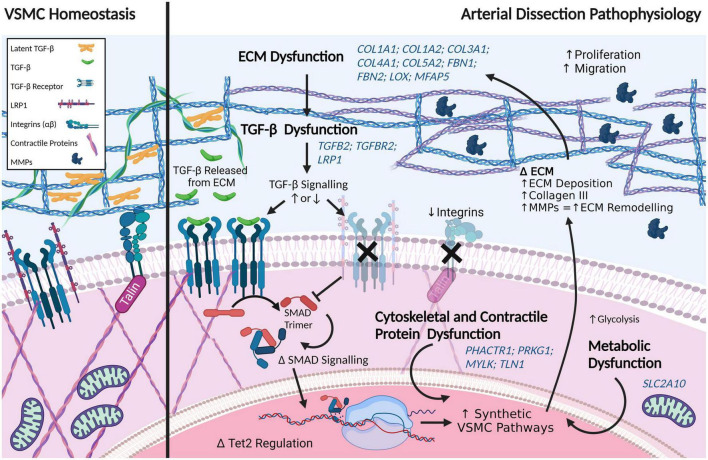
Pathways perturbed in arterial dissections include the extracellular matrix (ECM), TGF-β, signaling, cellular contraction/cytoskeleton, and metabolism. Dysregulation in any number of these pathways can drive vascular smooth muscle cells (VSMCs) toward a more synthetic phenotype. Variants in the same genes (italicized in blue) belonging to these pathways have been commonly identified in at least two types of arterial dissections. Created with BioRender.com.

**FIGURE 7:**
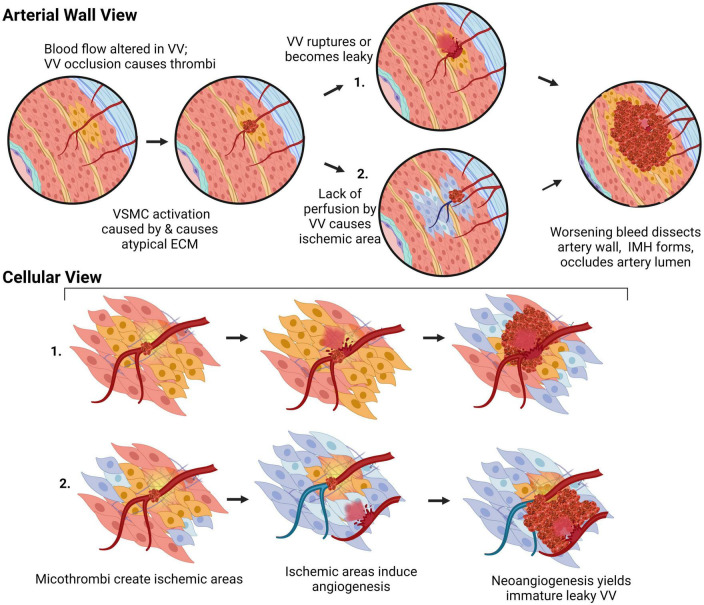
Proposed mechanism for arterial dissections. Atypical ECM deposition, activation of VSMC or EC will perturb vasa vasorum (VV) blood flow, leading to either their spontaneous rupture, or an area of ischemia encouraging growth of immature leaky vessels prone to bleed and, thus the development of an intramural hematoma, which impairs luminal blood flow resulting in tissue ischemia and/or infarction. Created with BioRender.com.

The commonalities link to mechanistic findings associated with arterial dissections. Perturbations in TGF-β signaling, the ECM, the cytoskeleton, and metabolism, are described in both spontaneous and syndromic dissections. In spontaneous dissections, variants in genes associated with these pathways including *PHACTR1, LRP1, SLC2A10, FBN1, COL3A1, COL4A1*, and *COL5A2* are common to at least two, if not three, arterial dissection subtypes (Figure 6 and Table 2). Importantly, dysfunction in these pathways is associated with vascular cell activation and subsequent phenotype switching. Phenotype switching has been described in aortic dissections (56, 117), but has not yet been a central theme in the pathophysiology of other arterial dissections. Nonetheless, we postulate that vascular cell phenotype switching can account for the pathology of arterial dissection: changes in vascular ECM can cause and be caused by cellular activation. Altered mechanical strength in the vasculature caused by altered VSMCs or ECM forces will likely affect vasa vasorum blood flow. Altered forces may cause occlusion of the vasa vasorum leading to ischemia and necrosis of the tunica media, particularly in arteries with low vasa vasorum density. Notably, vasa vasorum occlusion in porcine models has been shown to be sufficient to cause ischemia and dissection of the aorta (108), and reduced density of vasa vasorum inversely correlates with an increased risk of dissection in the coronary arteries (157, 159). Areas of ischemia will increase the likelihood of immune cell infiltration, which has been found in post-mortem arterial dissection tissues (222, 262). Moreover, areas of ischemia will, similar to atherosclerosis, stimulate neo-angiogenesis leading to proliferation of vasa vasorum. Histological evidence for increased abundance of vasa vasorum is reported in SCAD and CeAD (160, 222). This may be of significance since newly formed vasa vasorum lack a functional muscular layer (11) and are notoriously weak and leaky (263). Occlusion of vasa vasorum alone, or the growth of new still leaky vasa vasorum are potential sources of bleeding within the artery wall, IMH formation and vessel wall dissection. Similarly, endothelial phenotype switching from quiescent cells to activated phenotypes would weaken the endothelial barrier - in the intima or arteries and/or in the vasa vasorum, *per se* - again being a likely cause of bleeding and IMH formation (Figure 7). While more studies are needed to address these hypotheses, cellular activation represents a strong under-explored mechanism in arterial dissections, and, potentially, is an important therapeutic target.

**TABLE 2:** Common features of arterial dissections.

Features		Aortic dissections	SCAD	CeAD	Pulmonary
**Pathology**					
Intramural haematoma and intimal failure		(100)	(157)	(218)	(241)

Altered vasa vasorum		(108)	(160)	(222)	
**Risk factors**					

Sexual dimorphism		67.5% male age related dimorphism (109)	84% women (161)	57% male age related dimorphism (223)	Age related dimorphism (239)
Average age of dissection		63 years old (103)	51.1 years old (161)	45.3 years old (223)	44.8 years old (239)
Hypertension		(109)	(161)	(225)	(239, 242)
Migraine			(94, 188)	(227)	
Infection/Inflammation		(112)	(168)	(228)	Some evidence, (244)
Connective tissue disorders		(100)	(166)	(219)	(240)

**Examples of shared disease associated genes/pathways**					
	*COL1A1*	(*Limited data*) (132, 133)	(172);	(221)	
	*COL1A2*	(*Limited data*) (132)	(172, 185)	(221)	
	*COL3A1*	(121)	(172, 175, 185)	(234)	
ECM	*COL4A1*	(*Limited data*) (132)	(172, 185)	(230)	
	*COL5A2*	(125)	(172, 185)	(232)	
	*FBN1*	(121, 290)	(97, 172, 291)	(230)	
	*FBN2*	(121)	(172)		
	*LOX*	(135)	(175)		
	*MFAP5*	(134)	(172)	(221)	

TGF-β pathways	*LRP1*	(121, 126)	(97, 172, 188)	(233, 292)	
	*TGFBR2*	(121)		(230)	
	*TGFB2*	(119)	(Likely pathogenic) (175)		

Cytoskeletal/Contractile pathways	*PHACTR1*		(97, 171, 188)	(233)	
	*MYLK*	(121, 125)	(175)		
	*PRKG1*	(293)	(172)		
	*TLN1*	(142)	(203)		

VSMC metabolism	*SLC2A10*	(1)	(172)		

## Author contributions

MB and VR researched the literature and wrote the manuscript with input from KJ, EG, BM, JK, RL, SI, and RG. All authors contributed to the article and approved the submitted version.
